# Bowel strangulation caused by massive intraperitoneal adhesion due to effective chemotherapy for multiple peritoneal metastases originating from descending colon cancer

**DOI:** 10.1007/s12328-016-0679-y

**Published:** 2016-08-27

**Authors:** Nobutoshi Horii, Daisuke Morioka, Kazuya Yamaguchi, Yoshiki Sato, Masaru Miura, Mikiko Tanabe

**Affiliations:** 1 Department of Surgery, Yokohama Ekisaikai Hospital, 1-2 Yamadacho, Naka-ku, Yokohama, 231-0036 Japan; 2 Department of Pathology, Yokohama City University Medical Center, Yokohama, Japan

**Keywords:** Colorectal cancer, Peritoneal dissemination, Chemotherapy, Adhesion, Strangulated ileus

## Abstract

We describe a case of bowel strangulation caused by massive peritoneal adhesion as a result of effective chemotherapy. A 71-year-old man, who had obstructive descending colon cancer with massive peritoneal metastases and, therefore, received palliative surgery consisting of diverting colostomy and sampling of peritoneal nodules, developed bowel strangulation on day 4 of the 2nd course of chemotherapy, including irinotecan, l-leucovorin, and 5-fluorouracil. Emergent celiotomy showed a massive intraperitoneal adhesion formed around several intestinal loops, which were not observed at the prior surgery. One loop was strangled, but recovered by adhesiotomy alone. Intestinal loops were formed around aggregates of peritoneal nodules as the centers, several of which were then sampled. We closed the abdomen after all intestinal loops were eradicated by total enterolysis. Fortunately, the patient has been doing well and received chemotherapy without recurrent bowel obstruction 10 months after the present episode. Histological findings of the aggregates causing intestinal loops demonstrated extensive necrosis of cancerous tissue surrounded by fibrosis with abundant lymphocyte infiltration. These findings were not observed in the specimen sampled before chemotherapy, suggesting that intestinal loops were caused by inflammatory adhesion occurring around the peritoneal metastases as a result of effectiveness of chemotherapy.

## Introduction

Up to the 1990s, colorectal cancer patients with peritoneal metastases were considered terminally ill and, therefore, these patients generally received only best supportive care in many situations. Median survival time (MST) of these patients was reported to be approximately 6 months, and efficacy of chemotherapy for colorectal peritoneal metastases (CPM) was reported to be disappointing, with 7-9 months of gained MST [1–5]. According to the recent advancements in chemotherapeutic agents and/or regimens with biological targeted agents, however, survival outcomes of treatment for patients with unresectable colorectal cancer have been dramatically improved with more than 2 years of MST [4, 5]. In other words, application of cytotoxic chemotherapy with or without biologic targeted agents for patients with massive CPM, who might have been considered ineligible for chemotherapy in previous decades, seems currently common. Therefore, many adverse events which arise from implementation of chemotherapy have been increasingly reported and it is considered that these events would have not developed in previous decades when effective chemotherapeutic agents and/or regimens did not exist [5–7].

We herein report a case of bowel strangulation due to massive intraperitoneal adhesion as a result of effectiveness of chemotherapy for multiple peritoneal metastases originating from colorectal cancer.

## Case presentation

**Fig. 1:**
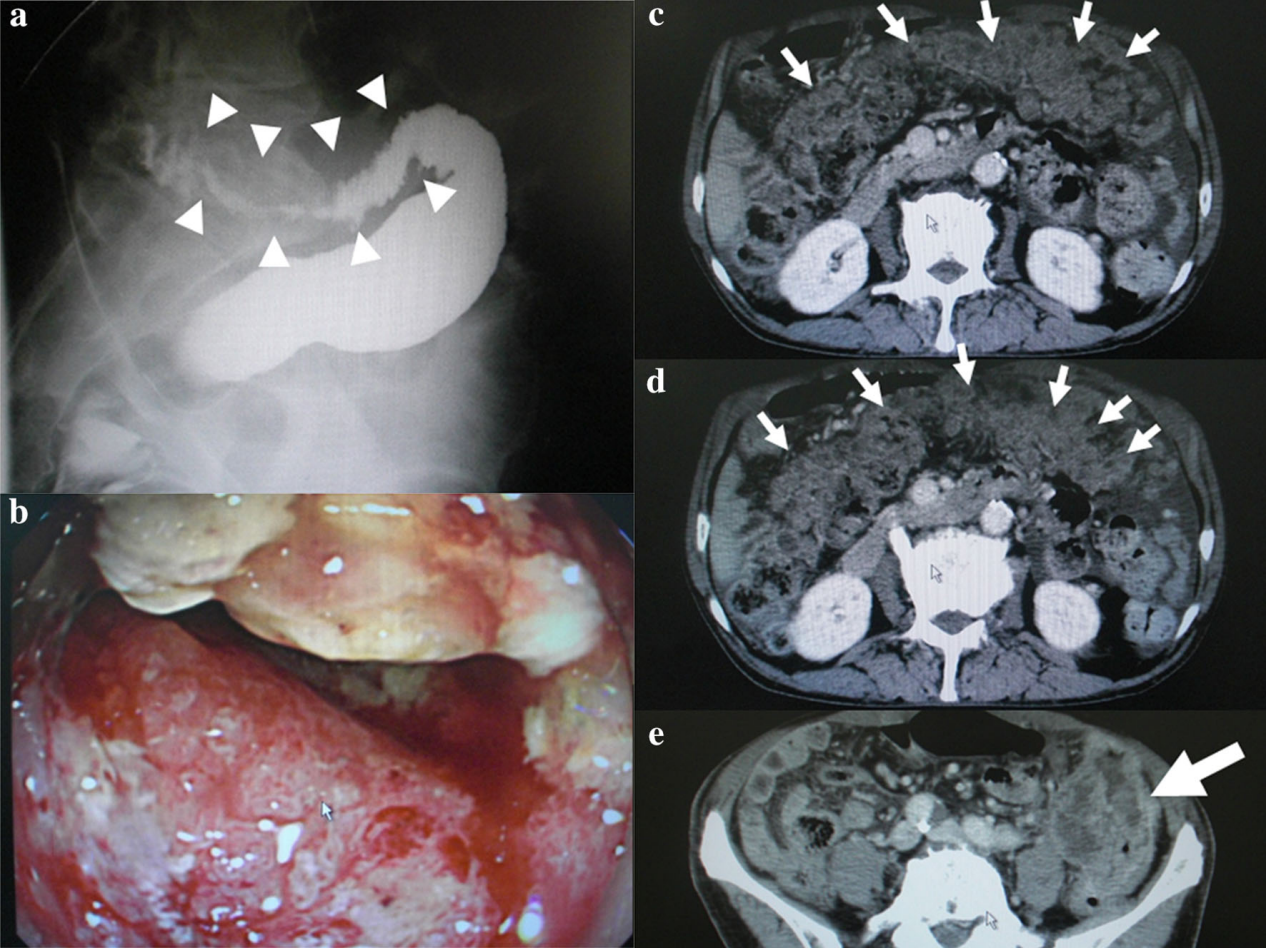
Findings of barium enema, colonoscopy, and abdominal computed tomography. **a** A severely stenotic lesion (*white arrowheads*) was observed in the descending colon just proximal to the sigmoid-descending junction. **b** A large circular type 2 lesion was detected by colonoscopy and caused severe bowel stenosis. **c**, **d** Greater omentum was occupied with numerous peritoneal nodules (*black arrows*) and formed an omental cake. Moderate ascites was identifiable. **e** Primary lesion was easily detectable as a large mass by computed tomography (*large white arrow*)

A 71-year-old man was seen at our hospital for complaints of abdominal fullness and a hard large tumor identified palpably in the left lower abdomen. He had a history of receiving surgery for rectal cancer, which was performed more than 10 years previously. After careful workups, descending colon cancer, causing bowel stenosis and multiple peritoneal metastases, was diagnosed (Fig. 1). Because bowel stenosis was severe, surgery was conducted in the semi-emergent setting. Intraabdominal findings showed that numerous peritoneal nodules and/or clusters of nodules were observed all over the abdomen and huge omental mass formed by peritoneal nodules occupied in the upper abdomen. Primary lesion was considered to be removable. However, R0 resection seemed impossible due to the massive peritoneal metastases accounting for Peritoneal Cancer Index [8] with 26 and Peritoneal Surface Disease Severity of Colon Cancer with Peritoneal Dissemination [9] with 11 points and Stage IV. Therefore, we decided to perform diverting colostomy and sampling peritoneal nodules. Because of huge omental mass, transverse colostomy was impractical. Thus, we mobilized the ascending colon for the colostomy and sampled several peritoneal nodules. Fortunately, intestinal obstruction was not observed other than the primary stenotic lesion in spite of massive peritoneal metastases at that time.

**Fig. 2:**
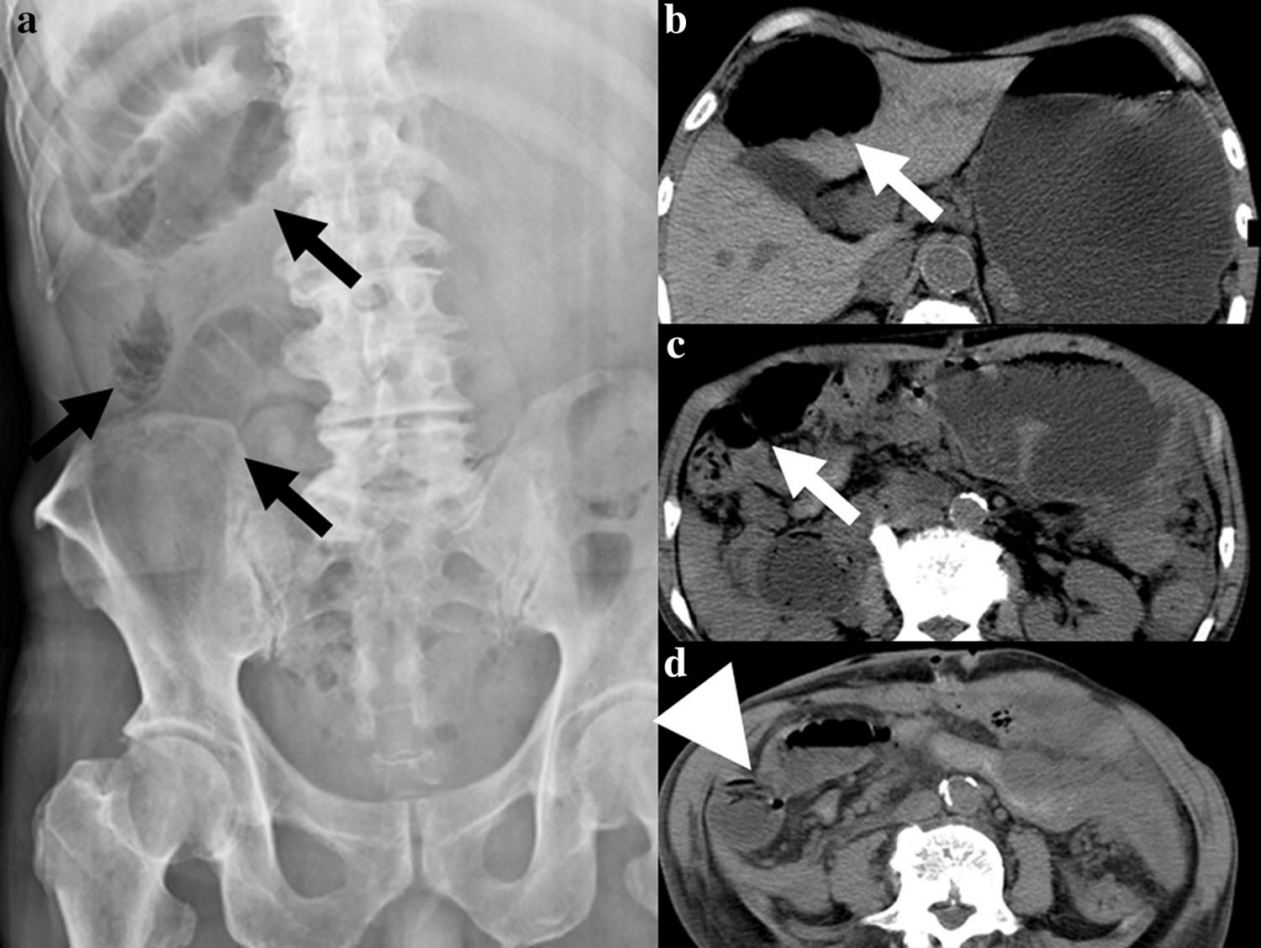
Findings of abdominal X-ray and computed tomography taken at the onset of bowel strangulation. **a** Abnormal intestinal gases (*black arrows*) observed by abdominal X-ray suggested formation of several intestinal loops. **b** Dilated intestine was observed in the right upper abdomen. **c**, **d** Dilatation of the intestine was tapered (*white arrow*) and disrupted by the severe caliber change (*white arrowhead*). These findings suggested that bowel strangulation was caused by the torsion of the intestinal loop

**Fig. 3:**
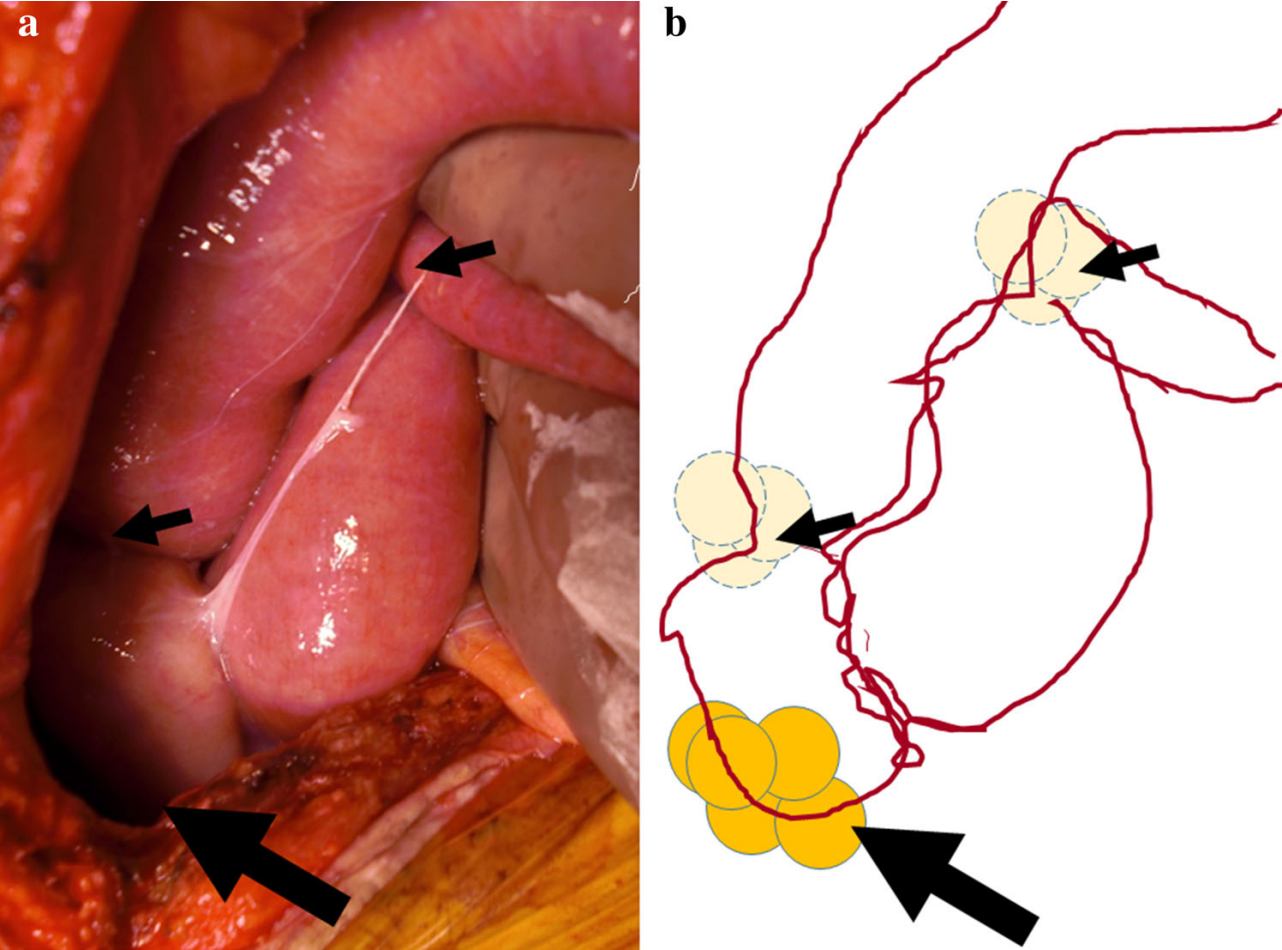
Photograph and schematic image of intraabdominal findings schematic image at the second emergent laparotomy. **a** Dilated and mildly discolored ileum was found in the right upper abdomen. **b** Intestinal loops were formed around the aggregates of peritoneal metastatic nodules as the centers (*small* and *large black arrows*). Notably, one loop was twisted on the aggregate at the axis (*large black arrows*) and strangled

Postoperative course was uneventful, and chemotherapy consisting of irinotecan, 5FU, and leucovorin (FOLFIRI) was started on postoperative day 7 and given biweekly. Any severe adverse effects, such as watery diarrhea, refractory constipation, or uncontrollable nausea, were not found other than mild anorexia until day 4 (25 days after surgery) of the 2nd course of FOLFIRI, when the patient developed sudden abdominal fullness causing epigastric pain and vomiting. Findings of abdominal X-ray and CT suggested intestinal obstruction (Fig. 2). Furthermore, strangulation of the intestine was strongly suspected based on the physical, laboratory, and imaging findings at that time. Hence, urgent celiotomy was conducted. The abdomen was easily opened because the intraabdominal organs scarcely adhered to the abdominal wall. Intraabdominal findings showed that the number and sizes of peritoneal nodules and/or clusters of nodules seemed markedly reduced. However, several intestinal loops were observed, and each loop was formed around a cluster of nodules at the center. One of the intestinal loops was mildly discolored by the strangulation (Fig. 3). Adhesions causative of these intestinal loops seemed not derived from prior surgical procedures because these intestinal loops did not adhere to the dissection surfaces, which were formed in prior surgery. Each nodule or cluster of nodules caused moderate to severe adhesion, but the adhesion was releasable with adhesiotomy alone although total enterolysis was necessary for eradicating all intestinal loops. After that, discolored intestine recovered normal color and normal peristalsis was observed throughout the abdomen. We washed the peritoneal cavity using warm saline and laid several sheets of bioresorbable hyaluronic acid/carboxymethylcellulose membrane [10] extensively in the abdomen. Then we closed the abdominal wall after sampling several peritoneal nodules, which caused intestinal loops. Fortunately, postoperative course was uneventful and the patient was discharged from hospital 7 days after the second urgent surgery and thereafter received chemotherapy again.

**Fig. 4:**
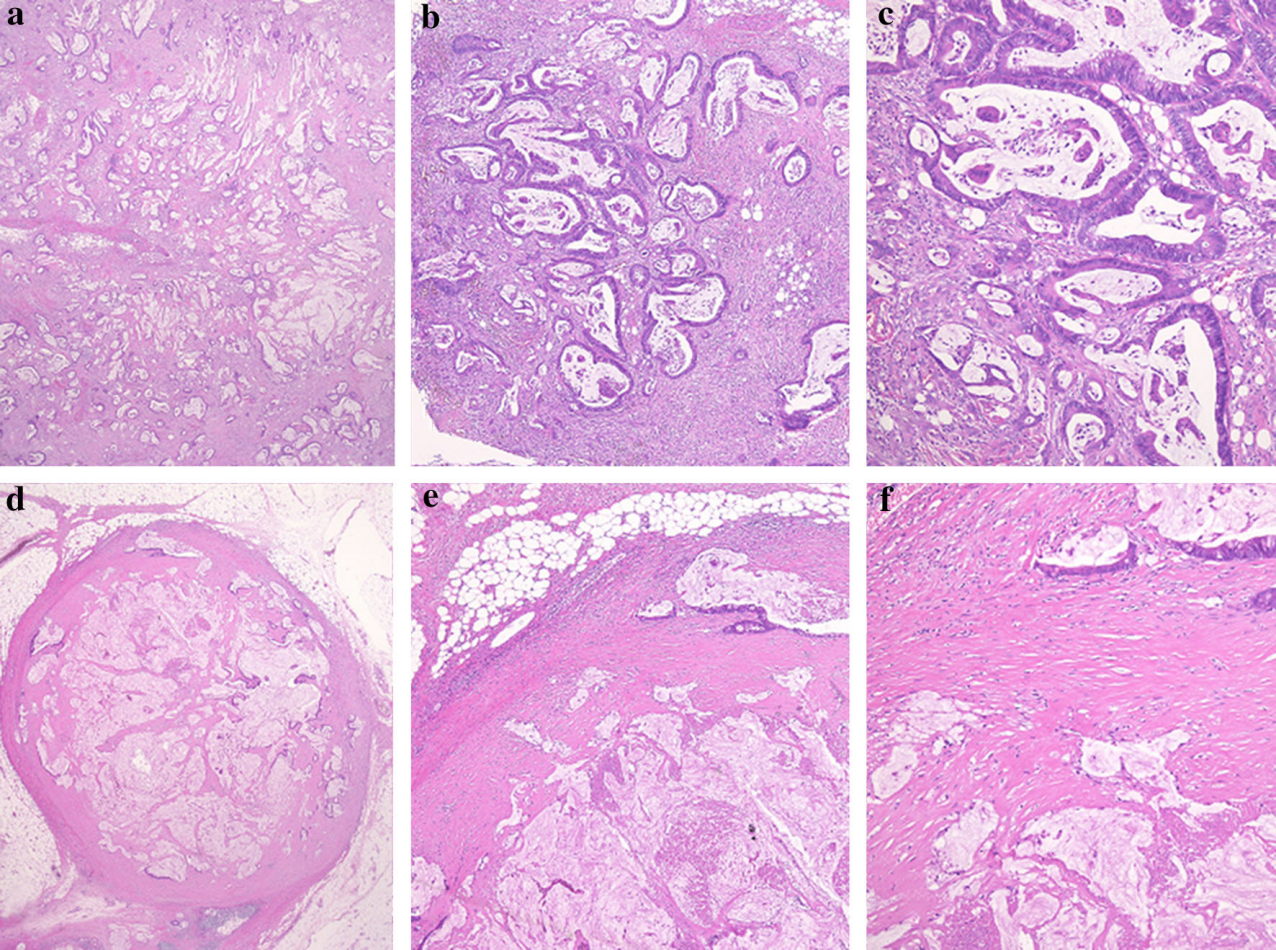
Comparison of histological findings between peritoneal nodules before and after chemotherapy. **a**–**c** Loupe image (**a** original magnification, ×5), low-power (**b** original magnification, ×40), and high-power maicroscopic findings (**c** original magnification, ×100) of the specimens sampled at the first semi-emergent surgey, i.e., before receiving chemotherapy, showed that peritoneal nodules were mainly composed of well-differentiated tubular adenocarcinoma and in part mucin-enriched cancerous cells, which leads to the diagnossis of well differentiated mucinous carcinoma. **d**–**f** Loupe image (**d** origincal magnification, ×5), low-power (**b**, **e** original magnification, ×40), and high-power maicroscopic findings (**f** original magnification, ×100) of the specimens sampled at emregent surgery for bowel strangulation, i.e., after two courses of chemotherapy, showed that peritoneal nodules were occupied mainly with abundant mucous lake and a little viable canceraous tissue was observed sparsely in the mucous lake. Each nodule was encapsulated with fresh fibrous tissue, through which mononuclear cell infiltration was diffusely observed. It seems that the fibrous tissue attached surrounding mesothelial membranous structures to peritoneal metastatic nodules

Histology findings of peritoneal nodules sampled at the second urgent surgery showed that cancerous tissue was nearly eradicated and fibrous tissue was formed surrounding the peritoneal nodules (Fig. 4). Furthermore, inflammatory cell infiltration, which was mainly composed of lymphocytes, was observed extensively in the fibrous tissue. In other words, each peritoneal metastatic nodule was encapsulated with fresh fibrous tissue, through which mononuclear cell infiltration was diffusely observed. It seems that surrounding intraabdominal structures were attched to each cluster of peritoneal metastatic nodules by the fibrous tissue. These findings were not observed in the peritoneal nodules sampled at the first celiotomy (Fig. 4), which was performed before chemotherapy, suggesting that formation of intestinal loops was caused by inflammatory adhesion occurring around the peritoneal metastases as a result of effectiveness of chemotherapy. Serum carcinoembryonic antigen and carbohydrate antigen 19-9 levels, both of which were measured before induction of chemotherapy and after the episode of bowel strangulation, reduced from 7.2 ng/ml to 1.0 ng/ml and from 48.8 IU/ml to 35.5 IU/ml, respectively. These results also supported the effectiveness of chemotherapy.

Fortunately, the patient has been doing well and receiving chemotherapy with targeted agent (bevacizumab), which was added from the 4th course, 10 months after the diagnosis of descending colon cancer without recurrent intestinal obstruction. At this stage, efficacy of chemotherapy was judged to be partial response of Response Evaluation Criteria in Solid Tumors version 1. 1 [11].

## Discussion

During last 15 years, markedly effective cytotoxic chemotherapies and biological targeted agents were developed and prognosis of patients with unresectable colorectal cancer has been dramatically improved. However, although peritoneal metastasis is the second most frequent distant metastasis of colorectal cancer following liver metastasis, treatment outcomes of patients with CPM were dismal in the previous decades when no effective drugs were present other than 5-fluorouracil [1–5, 7–10, 12]. Recently, new chemotherapeutic and targeted agents have improved the prognosis of these patients although treatment outcome seemed still unsatisfactory with systemic chemotherapy alone [7–10, 12]. Furthermore, cytoreductive surgery (CRS) with hyperthermal intraperitoneal chemotherapy (HIPEC) for CPM was introduced in the 1990s [13]. This aggressive approach for CPM has been gaining wider acceptance and thus being standardized [5, 7–10, 12]. Current stance of applying CPS with HIPEC for CPM has been established with effective chemotherapy as a prerequisite [5, 7–10, 12]. Therefore, indication of induction chemotherapy for CPM will expand and, therefore, application of chemotherapy accompanied by the standardization of CRS with HIPEC must be increasingly given for patients with CPM [5–7, 10, 12].

Based on the comparison of histological findings between peritoneal nodules sampled before and after the induction of chemotherapy in the present case, bowel strangulation was considered to result from the effect of chemotherapy, which was essentially beneficial, but not adverse. It was reported that tumor cell death induced intra- and/or peri-tumoral inflammatory cell infiltration, mainly composed of lymphocyte, for scavenging necrotic tumor tissue [14, 15]. In other words, the cause of massive peritoneal adhesion in the present case can be explainable as follows. Administration of chemotherapy caused necrosis of cancerous tissue and subsequent lymphocyte infiltration around the peritoneal metastatic nodules, which were intended to scavenge necrotic tumor tissue and consequently brought on inflammatory reaction surrounding the nodules [14, 15]. The inflammatory reaction formed fibrosis around the peritoneal metastases. In the present case, peritoneal metastases were severe and extended throughout the abdomen. Thus, inflammatory reaction caused by effectiveness of chemotherapy occurred throughout the abdomen and resulted in massive peritoneal adhesion. To the best of our knowledge, there has been no report describing this condition. Hence, we should consider this case to be unusual. However, in the modern era of effective chemotherapy, in which indication and application of chemotherapy have expanded, we must be aware that not only adverse effects, but also effectiveness of chemotherapy may lead to serious complications and, therefore, this condition, i.e., massive peritoneal adhesion as a result of effective chemotherapy, should be always taken into account when patients with multiple peritoneal metastases are treated by chemotherapy.

## Abbreviations

MST: Median survival time
CPM: Colorectal peritoneal metastases
FOLFIRI: Chemotherapy consisting of irinotecan, 5FU, and leucovorin
CRS: Cytoreductive surgery
HIPEC: Hyperthermal intraperitoneal chemotherapy
